# The therapeutic efficacy of transcranial direct current stimulation in managing Alzheimer’s disease: a systematic review and meta-analysis

**DOI:** 10.3389/fnagi.2026.1726469

**Published:** 2026-04-10

**Authors:** Xin Wang, Lu Tian

**Affiliations:** 1 Department of Neurosurgery, Shandong Provincial Hospital Affiliated to Shandong First Medical University, Jinan, Shandong, China; 2 Department of Neurosurgery, The First Affiliated Hospital of Shandong First Medical University & Shandong Provincial Qianfoshan Hospital, Jinan, Shandong, China; 3 Economic Operation Management Office, The First Affiliated Hospital of Shandong First Medical University & Shandong Provincial Qianfoshan Hospital, Jinan, Shandong, China

**Keywords:** Alzheimer’s disease, meta-analysis, randomized controlled trial, systematic review, therapeutic efficacy, transcranial direct current stimulation

## Abstract

**Objective:**

The present study aimed to investigate the therapeutic efficacy of transcranial direct current stimulation (tDCS) for Alzheimer’s disease (AD) and identify potential influential factors.

**Methods:**

A comprehensive literature search was conducted in PubMed, Embase, Web of Science, and the Cochrane Library up to April 2025. Eligible studies were randomized controlled trials (RCTs) in which tDCS was the sole differential intervention between study arms. The pooled effects of tDCS on patients’ global cognition, language, memory, executive function, and emotion were evaluated. Subgroup analyses were also performed to identify potential influential factors.

**Results:**

A total of 23 studies involving 24 trials and 823 mild to moderate AD patients were included. Our meta-analysis showed that tDCS significantly improved global cognition in AD patients (standardized mean difference [SMD] = 0.66; 95% confidence interval [CI], 0.38–0.95; *p* < 0.01), but had no significant effects on language or emotion. Subgroup analyses further revealed that significant memory improvement was observed in patients who received ≤ 10 sessions of tDCS and those with >6 years of education. Additionally, executive function was improved in patients who received stimulation on the left dorsolateral prefrontal cortex and in tDCS groups with ≤ 10 sessions. Moreover, improved executive function was observed in patients with 6–10 years of education, but not in other subgroups.

**Conclusion:**

tDCS treatment leads to improvements in global cognition, memory, and executive function in AD patients, but not in language or psychomotor symptoms. However, due to the relatively high heterogeneity of the included data, further well-designed studies are warranted before tDCS can be established as a standard therapeutic approach for AD.

## 1 Introduction

Alzheimer’s disease (AD) is a progressive neurodegenerative disorder and the most common cause of dementia in the elderly, posing a significant public health challenge globally. It is estimated that there are currently 44 million AD patients worldwide, with this number projected to reach 65.7 million by 2030 and triple by 2050 (Alzheimer’s Association, 2016). Clinically, AD is mainly characterized by global cognitive impairment, memory decline, executive dysfunction, disturbed psychomotor behaviors, and social withdrawal, which impose substantial burdens on families, healthcare systems, and society (Scheltens et al., 2016).

To date, there are mainly five types of FDA approved medications for the treatment of AD and AD-related dementia (Raina et al., 2008). These traditional agents, including donepezil, rivastigmine, galantamine, and memantine, have limited long-term efficacy and may induce certain side effects. Lecanemab, a novel humanized monoclonal antibody introduced in China in 2024, can selectively target soluble Aβ protofibrils and has been recognized as a promising disease-modifying therapy for early-stage AD (Liao et al., 2026; van Dyck et al., 2023). However, in addition to its high cost, Lecanemab may also cause adverse events such as infusion-related reactions, tremor, dizziness, headache, and elevated blood pressure (Liao et al., 2026). In contrast, transcranial direct current stimulation (tDCS), a non-invasive and low-cost intervention that does not require intravenous infusion, has shown potential in enhancing neuroplasticity and improving cognitive function in AD patients.

As a non-invasive brain stimulation technique (NIBS), tDCS delivers low electric current via electrodes positioned on the scalp. This current, typically ranging from 1 to 2 mA, generates a weak electric field within brain tissue, which modulates cerebral neuronal membrane potentials and thereby alters various aspects of cognition, emotion, and psychological status (Kidgell et al., 2013; Kuo et al., 2014; Nitsche et al., 2008). Compared with another NIBS technique, repetitive transcranial magnetic stimulation (rTMS), tDCS is equally safe, tolerable, and painless, while being more cost-effective and easier to operate (Majdi et al., 2022). Consequently, the application of tDCS in AD treatment has attracted growing attention in both clinical and academic fields.

Although an increasing number of original studies (Boggio et al., 2012; Boggio et al., 2009; Bystad et al., 2016; Cespon et al., 2019; Cotelli et al., 2014; Ferrucci et al., 2008; Gangemi et al., 2021; Gangemi and Fabio, 2020; Im et al., 2019; Khedr et al., 2014; Khedr et al., 2019; Lu et al., 2019; Smirni et al., 2021; Suemoto et al., 2014) and meta-analyses (Koo et al., 2023; Talar et al., 2022) have evaluated the efficacy and safety of tDCS in patients with AD and AD-related dementia, no consensus has been reached. For instance, several studies have reported cognitive improvements following tDCS intervention (Boggio et al., 2012; Boggio et al., 2009; Bystad et al., 2016; Cotelli et al., 2014; Ferrucci et al., 2008; Khedr et al., 2014), whereas others have failed to demonstrate significant effects (Suemoto et al., 2014). Although our previous study (Wang and Tian, 2024) preliminarily indicated that tDCS significantly improves cognitive function, the issue of optimal tDCS scheduling remains unresolved. Additionally, only global cognitive outcomes were assessed in that study. The meta-analysis of Majdi et al. (2022) investigated the effects of tDCS on various cognitive domains but was limited by a small number of included studies and high heterogeneity. Since then, several large, well-designed randomized controlled trials (RCTs) have been published (Andrade et al., 2022; Fonte et al., 2024; Hu et al., 2022; Kim and Yang, 2023; Li et al., 2023; LoBue et al., 2025; Martorella et al., 2023; Meléndez et al., 2023; Satorres et al., 2022), providing an opportunity to comprehensively evaluate the therapeutic efficacy of tDCS on different cognitive domains and psychomotor symptoms through an updated meta-analysis.

Therefore, we conducted this systematic review and meta-analysis based on all eligible RCTs published up to April 2025, aiming to draw a comprehensive conclusion on the therapeutic efficacy of tDCS in AD patients from multiple perspectives. We analyzed the effects of tDCS on global cognition, memory, language, executive function, and psychomotor status. Additionally, we performed subgroup analyses to compare different stimulation settings and identify the optimal candidates for tDCS intervention.

## 2 Methods

This study was performed in accordance with the Preferred Reporting Items for Systematic Reviews and Meta-Analyses (PRISMA) guidelines (Page et al., 2021) and the Cochrane Handbook for the Systematic review of Interventions (Shuster, 2011).

### 2.1 Literature search strategy and eligibility criteria

A comprehensive literature search was conducted in PubMed, Embase, Web of Science, and Cochrane Library up to April 2025 to identify relevant studies. The search strategy used the same keywords as in our previous work (Wang and Tian, 2024): (“Alzheimer disease” OR “Alzheimer’s disease” OR “Alzheimer dementia” OR “Alzheimer’s dementia” OR “AD”) AND (“transcranial direct current stimulation” OR “transcranial electrical stimulation” OR “tDCS” OR “tES”). Only parallel-arm and crossover randomized controlled trials (RCTs) were included, with no restrictions on publication date. All retrieved records were managed using EndNote X9 (Thompson ISI Research Soft, Philadelphia, PA, USA).

### 2.2 Eligibility criteria

Study selection was performed based on the PICOS (Population, Intervention, Comparison, Outcome, Study design) framework, as detailed below:

*Population*: Patients diagnosed with AD according to the criteria of the National Institute of Neurological and Communicative Disorders and Stroke/Alzheimer’s Disease and Related Disorders Association (NINCDS/ADRDA) (Dubois et al., 2007) or other standardized diagnostic criteria. At baseline, Mini-Mental State Examination (MMSE) scores was required to be ≥10 for literate patients and ≥8 for illiterate individuals.
*Intervention*: tDCS was administered to an age- and sex-neutral AD patient cohort.
*Comparison*: Anodal tDCS applied to specific cortical regions as the experimental intervention, and sham tDCS applied to the same cortical regions as the control condition.
*Outcomes*: Changes in global cognition and subdomains (memory, attention, language, executive function) and psychological symptoms from baseline to the first post-intervention assessment, measured using validated neuropsychological scales. Global cognition was mainly assessed by the MMSE or Alzheimer Disease Assessment Scale-cognitive subsection (ADAS-cog), and language function by the Verbal Fluency Task (VFT), among others. Outcome data were required to be reported as or convertible to mean ± standard deviation (SD).
*Study design*: Parallel-group and crossover RCTs.

Studies were excluded if they were: animal experiments, case reports, case series, non-RCTs, reviews, or studies involving non-AD dementia (e.g., frontotemporal dementia, Parkinson-related dementia); studies using other interventions (e.g., rTMS); or studies lacking sufficient data to calculate mean ± SD. Literature search and study screening were performed independently by two authors (XW and LT). Any discrepancies were resolved through discussion until consensus was reached.

### 2.3 Data extraction and outcome measures

Two authors independently extracted the following data from each included trial: first author, publication year, country, study design (parallel or crossover), sample size, patient characteristics (age, sex, disease duration, baseline cognitive and psychological measures), tDCS stimulation parameters (current density, number of sessions, stimulation duration, concurrent cognitive training), and outcomes of interest (post-treatment scale scores relative to baseline). If multiple RCTs were reported in a single article, each was treated as an independent trial. Adverse events and complications were also documented. Disagreements during data extraction were resolved via consensus discussion.

In this meta-analysis, mean changes and SD changes from baseline to post-intervention were calculated for each outcome. As described in previous studies (Wang et al., 2020a; Wang et al., 2020b; Wang and Tian, 2024), if these change values were not directly reported, the following formulas were used:


$$\mathrm{SD}\;\text{Change}=\frac{\begin{array}{l} \text{Mean change}=\text{Mean final}-\\ \text{Mean baseline} \end{array}}{\sqrt{\mathrm{SD}\;\text{baselin}e^{2}+\mathrm{SD}\;\text{fina}l^{2}-}(\begin{array}{l} 2\times \text{coeffiecint}\times \\ \mathrm{SD}\;\text{baseline}\times \mathrm{SD}\;\text{final} \end{array})}$$

For studies reporting mean ± standard error (SE), SD was converted using: SD = SE× √n (*n* denotes the sample size). Studies for which these conversions could not be performed were excluded from the quantitative synthesis. If multiple time points were reported, only data from the first post-intervention assessment were extracted.

### 2.4 Research quality and risk of bias assessment

Two reviewers independently assessed the methodological quality and risk of bias of included studies using the Cochrane Collaboration Risk of Bias 2 (RoB2) tool (Sterne et al., 2019). The following domains were evaluated: random sequence generation and allocation concealment (selection bias), blinding of participants and personnel (performance bias), blinding of outcome assessment (detection bias), incomplete outcome data (attrition bias), selective reporting (reporting bias), and other potential sources of bias. A risk-of-bias plot was used for visual presentation. Discrepancies were resolved by discussion.

### 2.5 Certainty of evidence

The Grading of Recommendations Assessment, Development, and Evaluation (GRADE) approach was used to assess the certainty of evidence for each outcome. GRADE ratings consider five domains: risk of bias, inconsistency, indirectness, imprecision, and other relevant factors. The overall quality of evidence was classified as high, moderate, low, or very low.

### 2.6 Data analysis

The primary outcome was the pooled effect of tDCS on global cognition, language, memory, executive function, and psychological status in patients with AD. We used data obtained immediately after tDCS or at the first available assessment (usually within 1 day) if immediate data were not reported. Because different assessment scales were used across studies, standardized mean difference (SMD) with 95% confidence interval (CI) was adopted to pool effect sizes. Between-study heterogeneity was evaluated using the Cochran Q statistic *p*-value and *I*^2^ statistic. An I^2^ ≥ 50% indicated substantial heterogeneity, and a random-effects model was applied. Given the expected clinical and methodological diversity across included studies, both random-effects and fixed-effects models were used for all analyses, even when I^2^ < 50%.

Subgroup analyses were performed to explore potential sources of heterogeneity based on: stimulation regions (L-DLPFC *vs.* temporal lobe [TL]), session numbers (˃10 *vs*. ≤10), duration of stimulation (≥25 min *vs.* <25 min), current density (<0.08 mA/cm^2^ *vs.* ≥0.08 mA/cm^2^), years of education (˃10 *vs.* ˃6 and ≤10 *vs.* ≤6), as well as concurrent cognitive training (CT, yes *vs*. no). These subgroup definitions were consistent with our previous study (Wang and Tian, 2024).

All statistical analyses were performed using the “meta” and “forestplot” packages in R software (version 4.4.3, R Foundation for Statistical Computing, Vienna, Austria).

## 3 Results

### 3.1 Literature screening

**Figure 1:**
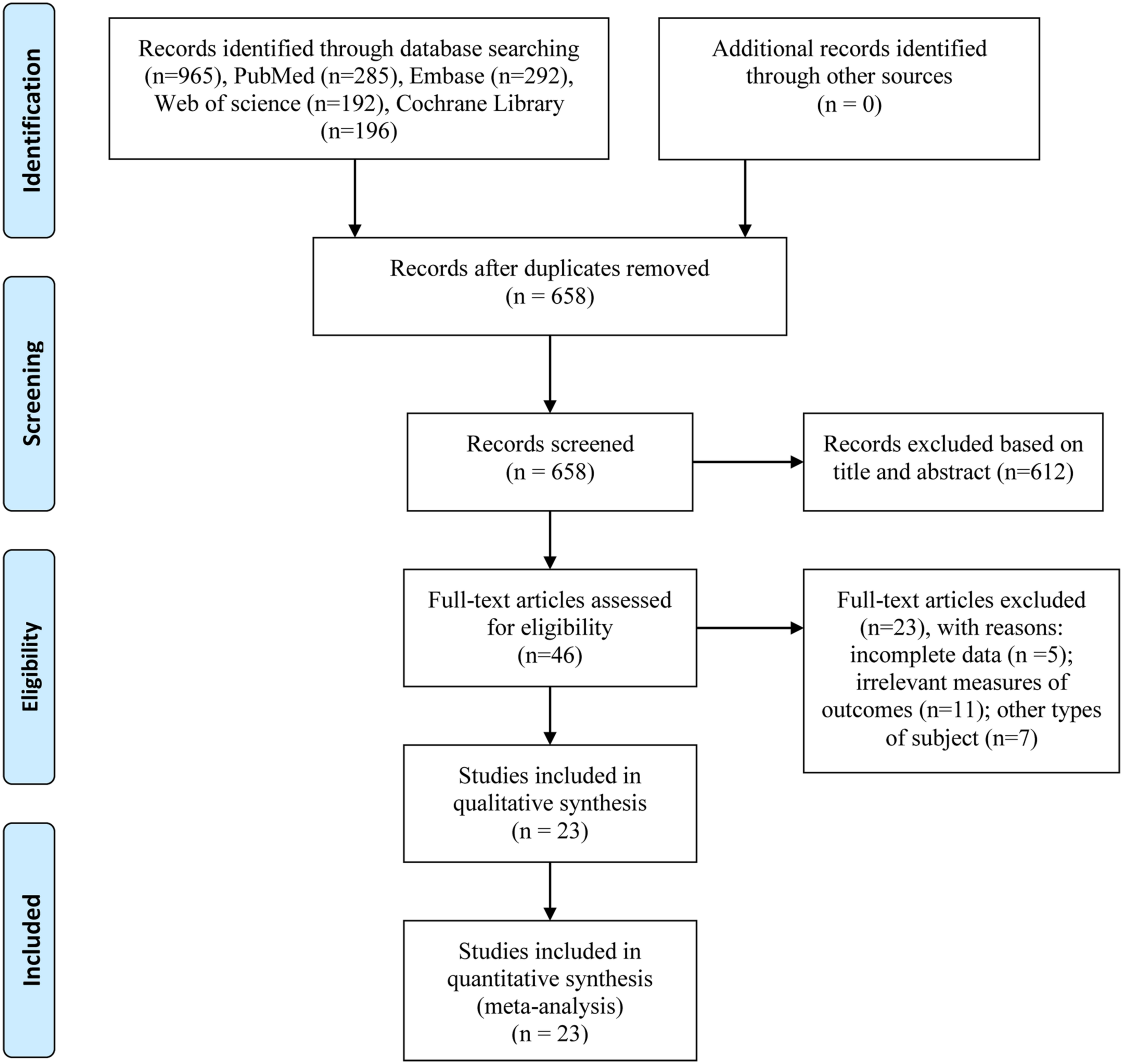
Flow diagram in accordance with PRISMA statement (www.prisma-statement.org).

The study selection process is illustrated in Figure 1. A total of 965 articles were identified from the four major databases. Among these, 307 studies were excluded due to duplication, leaving 658 articles for title and abstract screening. Additional studies were excluded for reasons including irrelevant topics, basic research, or non-RCT designs. A total of 46 studies proceeded to full-text assessment, of which 5 were excluded due to insufficient data (*n* = 2), 11 due to irrelevant outcome measures, and 7 due to involving other types of dementia. Finally, 23 articles were included in the systematic review and meta-analysis, with publication dates ranging from August 2008 to January 2025. The PRISMA 2020 for abstracts checklist was shown in Supplementary Table S1.

### 3.2 Basic information of included studies

The basic characteristics of the included articles are summarized in Table 1. A total of 23 studies reporting 24 RCTs involving 823 patients diagnosed with mild to moderate AD were included in the meta-analysis (Andrade et al., 2022; Boggio et al., 2012; Boggio et al., 2009; Bystad et al., 2016; Cespon et al., 2019; Cotelli et al., 2014; Ferrucci et al., 2008; Fonte et al., 2024; Gangemi et al., 2021; Gangemi and Fabio, 2020; Hu et al., 2022; Im et al., 2019; Khedr et al., 2014; Khedr et al., 2019; Kim and Yang, 2023; Li et al., 2023; LoBue et al., 2025; Lu et al., 2019; Martorella et al., 2023; Meléndez et al., 2023; Satorres et al., 2022; Smirni et al., 2021; Suemoto et al., 2014). The follow-up periods in the included articles ranged from 1 to 6 months after the final tDCS session. Eighteen studies adopted a parallel RCT design, while only 4 studies reported crossover RCTs (Boggio et al., 2012; Boggio et al., 2009; Cespon et al., 2019; Ferrucci et al., 2008). One article (Gangemi et al., 2021) reported two parallel RCTs, which used the same tDCS protocol except for the number of sessions; all other studies reported a single trial. For studies that divided patients into anodal tDCS, cathodal tDCS, and sham stimulation arms (Cotelli et al., 2014; Khedr et al., 2014; Lu et al., 2019), only data from the anodal and sham stimulation arms were included in the meta-analysis.

**Table 1:** Characteristics of the 23 studies included in the systematic review and meta-analysis.

Study	Design	Sample size (tDCS/Sham)	Mean age (y) (tDCS/Sham)	Sex (M/F)	Education (tDCS/Sham)	Evaluated domains	Stimulation settings	Stimulation sites
Ferrucci et al. (2008)	Crossover	10 (10/10)	75.2/75.2	3/7	10.9/10.9	Memory and attention	1.5 mA, 15 min, single session	L,R-TPR
Boggio et al. (2009)	Crossover	15 (15/15)	77.5/77.5	8/7	13.3/13.3	Memory	2 mA (0.06 mA/cm^2^); 30 min/d for 5 days	L,R-TC
Boggio et al. (2012)	Crossover	15 (15/15)	77.5/77.5	8/7	13.3/13.3	General cognition, memory, and execution	2 mA (0.06 mA/cm^2^); 30 min/d for 5 days	L,R-TC
Cotelli et al. (2014)	Parallel	24 (12/12)	76.6/74.7	5/19	5.5/8.9	General cognition, memory, execution, and emotion	2 mA (0.08 mA/cm^2^); 25 min/d for 10 days	L-DLPFC
Khedr et al. (2014)	Parallel	22 (11/11)	68.5/67.3	11/11	NR	General cognition	2 mA (0.08 mA/cm^2^); 25 min/d for 10 days	L-DLPFC
Suemoto et al. (2014)	Parallel	40 (20/20)	79.4/81.6	12/28	5.0/4.5	General cognition, memory, language, and emotion	2 mA (0.06 mA/cm^2^); 20 min/d, 6 sessions	L-DLPFC
Bystad et al. (2016)	Parallel	25 (12/13)	70.0/75.0	14/11	NR	Memory	2 mA (0.06 mA/cm^2^); 20 min/d, 10 sessions	L-TC
Im et al. (2019)	Parallel	18 (11/7)	71.9/74.9	3/15	6.3/5.4	General cognition, memory, language, and execution	2 mA (0.06 mA/cm^2^); 30 min/d for 6 months	L-DLPFC
Khedr et al. (2019)	Parallel	44 (23/21)	64.2/65.2	26/18	4.0/3.5	General cognition, and emotion	2 mA (0.06 mA/cm^2^); 40 min/d, 10 sessions	L,R-TPR
Lu et al. (2019)	Parallel	133 (69/64)	74.2/74.5	38/78	7.3/6.5	General cognition, memory, language, and emotion	2 mA (0.06 mA/cm^2^); 20 min/d, 12 sessions	L-TC
Cespon et al. (2019)	Crossover	12 (12/12)	76/76	5/7	NR	Memory	1.5 mA, 13 min, single session	L-DLPFC
Gangemi and Fabio (2020)	Parallel	26 (15/11)	74/75	11/15	6.9/6.5	General cognition	2 mA (0.06 mA/cm^2^); 20 min/d, 10 sessions	L,R-DLPFC, L-TC
Gangemi et al. (2021)	Parallel	26 (13/13)^a^	67.5/69.0	10/16	6.5/6.1	General cognition	2 mA (2.5 mA/cm^2^); 25 min/d for 10 days 2 mA (2.5 mA/cm^2^); 25 min/d, 10d/m, 8 months	L-FTC
		18 (9/9)^b^	68.5/68.7	5/13	6.7/6.2			
Smirni et al. (2021)	Parallel	30 (10/20)	73.4/73.0	6/14	12.8/12.7	Language	1 mA, 30 min, single session	L-DLPFC
Andrade et al. (2022)	Parallel	36 (18/18)	75.4/77.1	19/17	4.4/5.6	General cognition	2 mA (0.08 mA/cm^2^); 30 min/d, 24 sessions	L,R-DLPFC
Hu et al. (2022)	Parallel	42 (21/21)	77.1/75.3	18/24	10.9/11.2	General cognition	2 mA; 30 min/d, 3 sessions/w,4 weeks	L,R-AG
Satorres et al. (2022)	Parallel	33 (17/16)	76.6/73.4	17/16	10.4/10.8	General cognition, memory, and execution	2 mA (0.08 mA/cm^2^); 20 min/d, 10 days	L-DLPFC
Martorella et al. (2023)	Parallel	40 (20/20)	74.2/71.9	11/29	NM	Emotion	2 mA (0.06 mA/cm^2^); 20 min/d, 5 sessions	R-supraorbital area
Kim and Yang (2023)	Parallel	16 (8/8)	71.1/69.9	9/7	13.5/14.4	General cognition, memory, and execution, and emotion	2 mA (0.06 mA/cm^2^); 20 min/d, 5 sessions	L-DLPFC
Li et al. (2023)	Parallel	124 (63/61)	76.7/45.6	41/83	NA	General cognition	2 mA (0.08 mA/cm^2^); 20 min/d, 60 sessions	L-DLPFC
Meléndez et al. (2023)	Parallel	18 (9/9)	73.7/75.4	7/11	7.9/8.9	General cognition and memory	2 mA (0.08 mA/cm^2^); 20 min/d, 5 sessions	L-DLPFC
Fonte et al. (2024)	Parallel	23(13/10)	81.2/85.2	9/14	8/8	General cognition, language, execution, and emotion	2 mA (0.08 mA/cm^2^); 15 min/d, 10 sessions	L-DLPFC
Lobue et al. (2025)	Parallel	33 (17/16)	76.6/73.4	17/16	10.4/10.8	Memory, language, and execution	2 mA (0.08 mA/cm^2^); 20 min/d, 10 days	L-DLPFC

stim, tDCS group; sham, sham group; MMSE, Mini-Mental State Examination; L, left; R, right; DLPFC, dorsolateral prefrontal cortex; TC, temporal cortex; TPR, temporoparietal region; FTC, frontotemporal cortex; AG, angular gyrus; d, day; w, week; m, month.

a Patients receiving tDCS for 10 days.

b Patients receiving tDCS for 8 months.

Regarding tDCS protocols, most studies selected the left dorsolateral prefrontal cortex (L-DLPFC) as the stimulation target (Cespon et al., 2019; Cotelli et al., 2014; Fonte et al., 2024; Im et al., 2019; Khedr et al., 2014; Kim and Yang, 2023; Li et al., 2023; LoBue et al., 2025; Meléndez et al., 2023; Satorres et al., 2022; Smirni et al., 2021; Suemoto et al., 2014), while other studies used the temporal cortex (TC) (Boggio et al., 2012; Boggio et al., 2009; Bystad et al., 2016; Gangemi and Fabio, 2020; Lu et al., 2019), temporoparietal region (TPR) (Ferrucci et al., 2008; Khedr et al., 2019), angular gyrus (AG) (Hu et al., 2022), frontal-temporal cortex (FTC) (Gangemi et al., 2021) and supraorbital area (Martorella et al., 2023). Different current densities were used across trials: 10 studies applied a current density of 0.06 mA/cm^2^ (Boggio et al., 2012; Boggio et al., 2009; Bystad et al., 2016; Gangemi and Fabio, 2020; Im et al., 2019; Khedr et al., 2019; Kim and Yang, 2023; Lu et al., 2019; Martorella et al., 2023; Suemoto et al., 2014), while 8 studies used 0.08 mA/cm^2^ (Andrade et al., 2022; Cotelli et al., 2014; Fonte et al., 2024; Khedr et al., 2014; Li et al., 2023; LoBue et al., 2025; Meléndez et al., 2023; Satorres et al., 2022). Most included studies applied repeated tDCS sessions, with the number of sessions ranging from 5 to 180, while only three studies used a single session (Cespon et al., 2019; Ferrucci et al., 2008; Smirni et al., 2021). Additionally, the duration of each stimulation session ranged from 15 to 40 min across the included trials.

### 3.3 Effects of tDCS on global cognition

**Figure 2:**
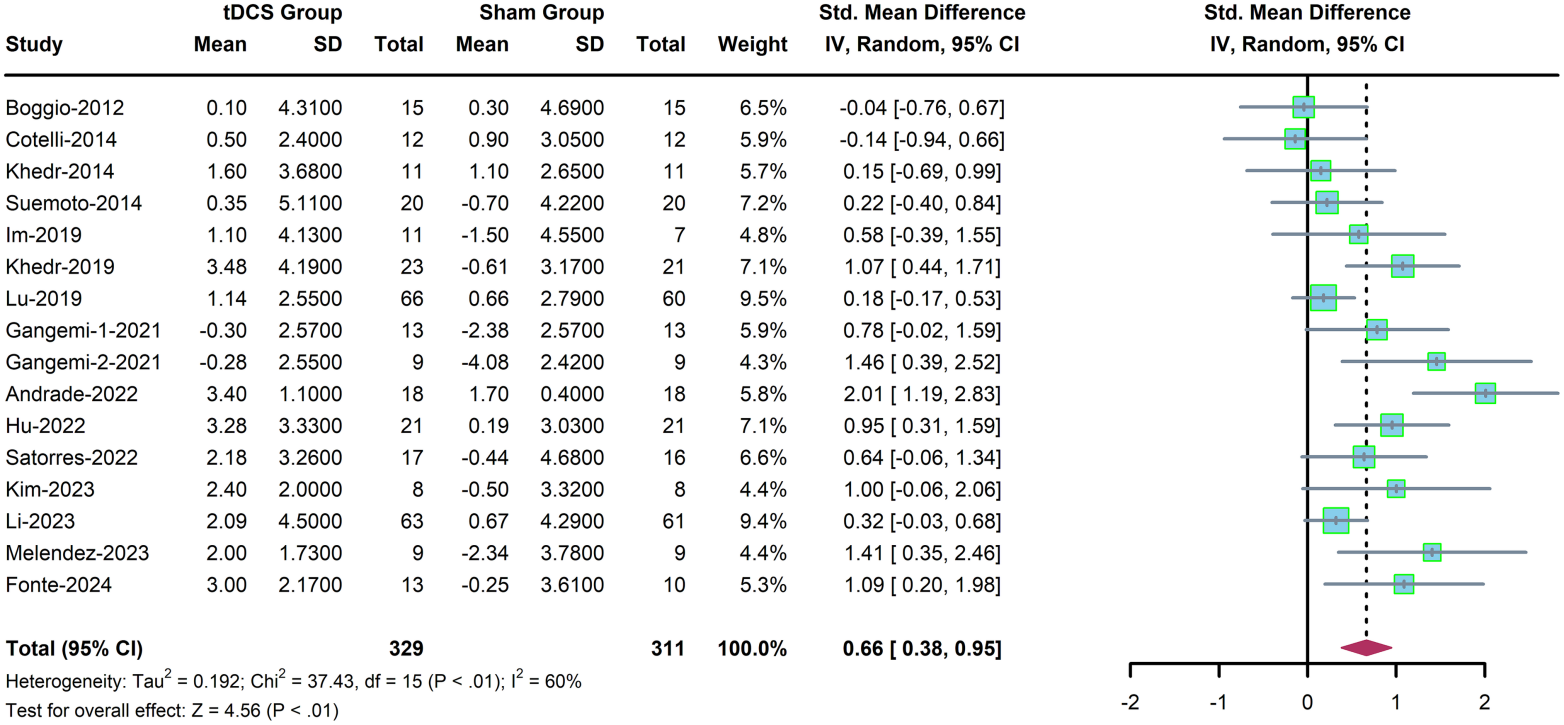
Forest plot for global cognition.

Sixteen trials were included to evaluate the efficacy of tDCS on global cognitive function in patients with AD. A random-effects model was used due to significant heterogeneity among the included studies (*p* < 0.01, *I*^2^ = 60%, Figure 2). The results showed that tDCS significantly improved global cognition in AD patients compared with sham stimulation (SMD, 0.66; 95% CI, 0.38–0.95; *p* < 0.01).

As shown in Table 2, subgroup analyses were performed to identify potential influential factors on global cognitive function. Consistent effects of tDCS were observed across all subgroups, including those stratified by stimulation site, number of sessions, stimulation duration, current density, years of education, and concurrent cognitive training.

**Table 2:** Results for subgroup analysis of general cognition.

Subgroup	No. trials	Sample size (tDCS/Sham)	SMD (95% CI)	Heterogeneity (*I*^2^) (%)	*p* value
Stimulation sites					
**TL**	**5**	**126/118**	**0.60 [0.09, 1.11]**	**66**	**0.02**
**L-DLPFC**	**9**	**164/154**	**0.47 [0.20, 0.74]**	**21**	**0.0008**
No. Sessions					
**Sessions ˃10**	**7**	**196/184**	**0.83 [0.36, 1.30]**	**74**	**0.0006**
**Sessions ≤10**	**9**	**133/127**	**0.54 [0.20, 0.88]**	**44**	**0.002**
Stimulation time (min)					
**High (≥25 min)**	**5**	**123/116**	**0.95 [0.26, 1.63]**	**80**	**0.007**
**Low (<25 min)**	**11**	**206/195**	**0.54 [0.25, 0.83]**	**43**	**0.0002**
Current density (mA/cm^2^)					
**<0.08**	**7**	**206/192**	**0.38 [0.12, 0.65]**	**33**	**0.005**
**≥0.08**	**8**	**102/98**	**0.89 [0.39, 1.40]**	**64**	**0.0005**
Years of education					
**˃10**	**5**	**124/121**	**0.50 [0.15, 0.85]**	**33**	**0.005**
**˃6 and ≤10**	**6**	**122/113**	**0.69 [0.17, 1.21]**	**63**	**0.009**
**≤ 6**	**4**	**72/66**	**0.96 [0.20, 1.71]**	**76**	**0.01**
Cognitive interventions					
**No**	**12**	**220/211**	**0.56 [0.37, 0.76]**	**34**	**0.0000**
**Yes**	**3**	**96/90**	**0.38 [0.08, 0.68]**	**89**	**0.01**

SMD, standardized mean difference; DLPFC, dorsolateral prefrontal cortex; TL, temporal lobe.

Lines in bold are statistically significant.

### 3.4 Efficacy of tDCS on language

**Figure 3:**
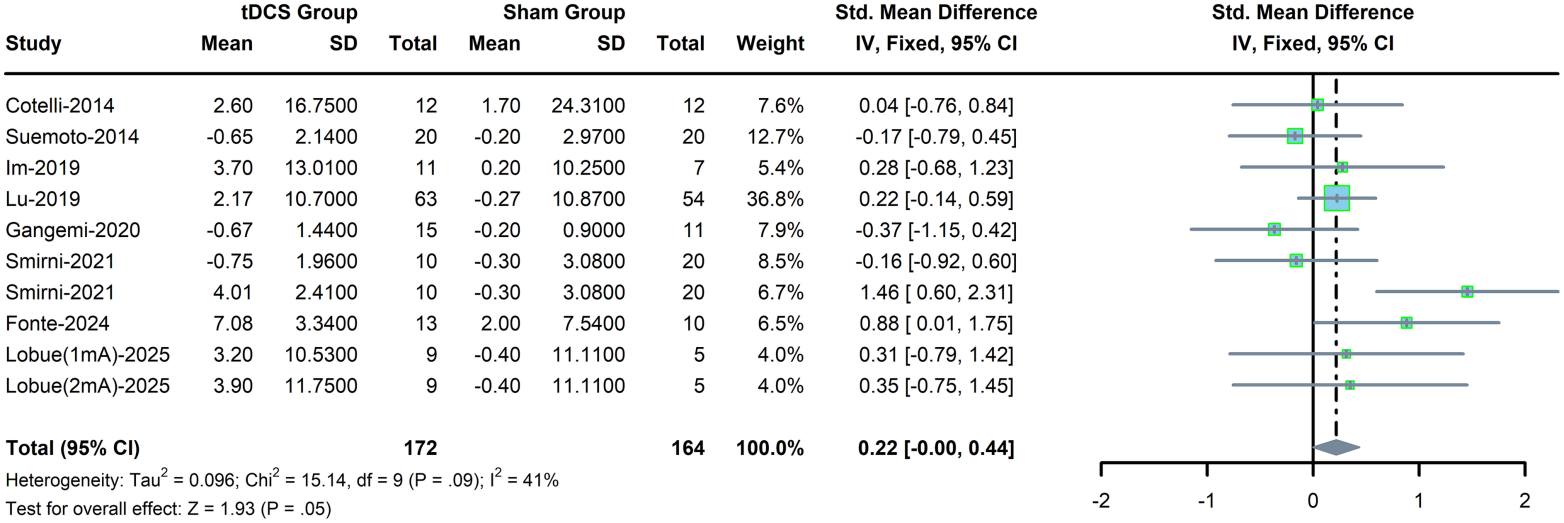
Forest plot for language ability.

Ten trials were included to assess the pooled effects of tDCS on language ability in AD patients. A fixed-effects model was adopted due to mild heterogeneity (*p* = 0.09, *I*^2^ = 41%, Figure 3). The results did not show a significant difference in language ability between the tDCS group and the sham stimulation group (SMD, 0.22; 95% CI, 0.00–0.44; *p* = 0.05). To test the reliability of the pooled effect, a random-effects model was also applied, and the effect size was consistent (data not shown). As shown in Table 3, the results of all subgroup analyses were similar to the main findings.

**Table 3:** Results for subgroup analysis of language.

Subgroup	No. trials	Sample size (tDCS/Sham)	SMD (95% CI)	Heterogeneity (*I*^2^) (%)	*p* value
Stimulation sites					
TL	1	63/54	0.22 [−0.44, 0.59]	-	0.23
L-DLPFC	5	66/69	0.10 [−0.25, 0.45]	10	0.58
No. Sessions					
Sessions ˃10	4	92/71	0.25 [−0.06, 0.56]	0	0.12
Sessions ≤10	6	80/93	0.25 [−0.30, 0.80]	67	0.37
Stimulation time (min)					
High (≥25 min)	2	23/19	0.14 [−0.47, 0.75]	0	0.66
Low (<25 min)	8	149/145	0.27 [−0.11, 0.66]	53	0.17
Current density (mA/cm^2^)					
<0.08	6	129/132	0.18 [−0.26, 0.62]	60	0.42
≥0.08	2	25/22	0.44 [−0.38, 1.27]	49	0.29
Years of education					
˃10	4	38/50	0.49 [−0.27, 1.25]	62	0.20
˃6 and ≤10	4	103/87	0.19 [−0.22, 0.59]	34	0.37
≤6	2	31/27	−0.04 [−0.56, 0.48]	0	0.89
Cognitive interventions					
No	7	84/88	0.20 [−0.26, 0.67]	52	0.39
Yes	2	75/66	0.19 [−0.14, 0.53]	0	0.25

SMD, standardized mean difference; DLPFC, dorsolateral prefrontal cortex; TL, temporal lobe.

### 3.5 Efficacy of tDCS on memory

**Figure 4:**
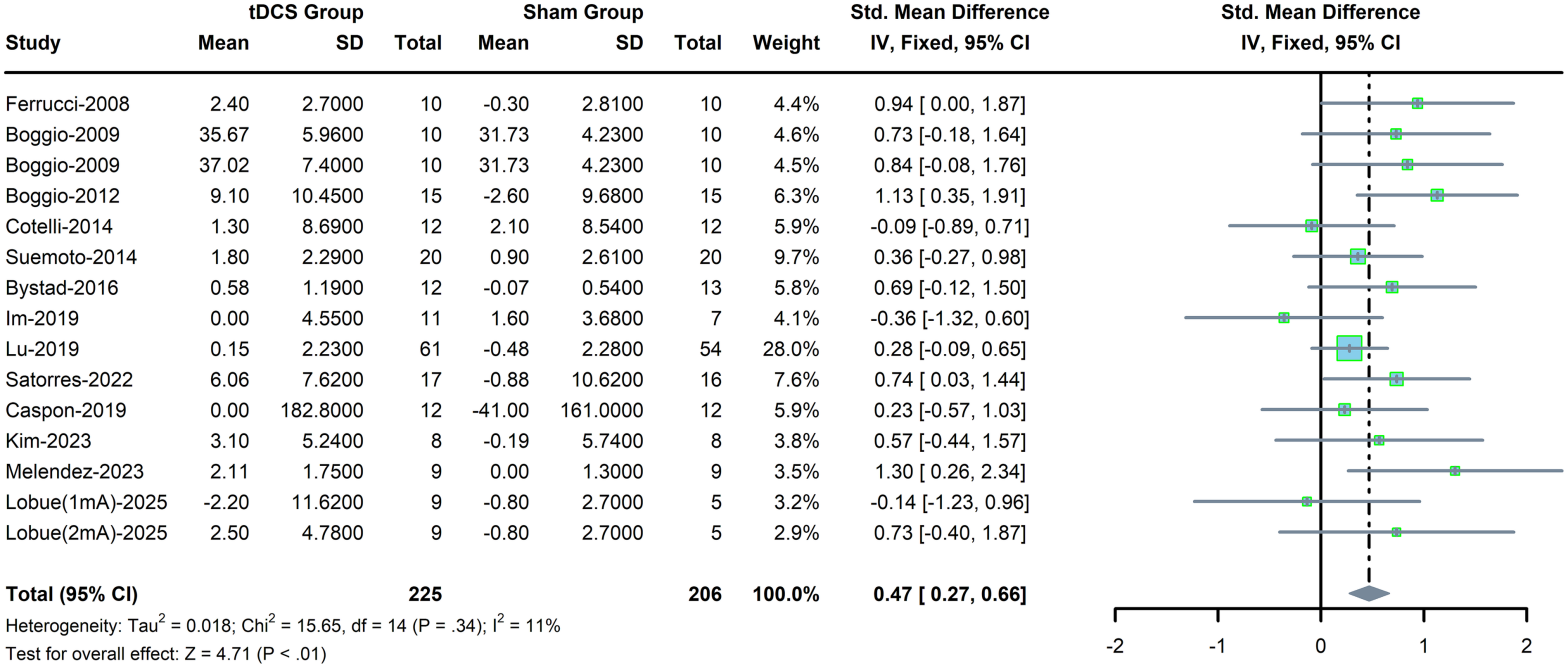
Forest plot for memory.

Fifteen RCTs were included to evaluate the efficacy of tDCS on memory in AD patients. A fixed-effects model was used due to mild heterogeneity (*p* = 0.34, *I*^2^ = 11%, Figure 4). The meta-analysis results indicated that the tDCS group had a significant improvement in memory compared with the sham stimulation group (SMD, 0.47; 95% CI, 0.27–0.66; *P*<0.01).

The results of subgroup analyses are shown in Table 4. Memory was significantly enhanced by ≤10 sessions of tDCS (SMD, 0.63; 95% CI, 0.38–0.89; *p* = 0.0000) but not by >10 sessions (SMD, 0.24; 95% CI, −0.06 to 0.54; *p* = 0.12). Furthermore, tDCS alone produced a significant improvement in memory (SMD, 0.60; 95% CI, 0.36–0.84; *p* = 0.0000), whereas tDCS combined with CT did not (SMD, 0.21; 95% CI, −0.12 to 0.55; *p* = 0.21). In addition, memory improvement was observed in patients with >6 years of education but not in other education subgroups. Consistent effects were observed in subgroup analyses stratified by stimulation site, stimulation duration, and current density.

**Table 4:** Results for subgroup analysis of memory.

Subgroup	No. trials	Sample size (tDCS/Sham)	SMD (95% CI)	Heterogeneity (*I*^2^) (%)	*p* value
Stimulation sites					
**TL**	**5**	**108/102**	**0.55 [0.27, 0.82]**	**26**	**0.0001**
**L-DLPFC**	**8**	**99/94**	**0.41 [0.12, 0.70]**	**17**	**0.006**
No. Sessions					
Sessions ˃ 10	5	98/79	0.24 [−0.06, 0.54]	0	0.12
**Sessions ≤10**	**10**	**127/127**	**0.63 [0.38, 0.89]**	**0**	**0.0000**
Stimulation time (min)					
**High (≥25 min)**	**6**	**70/67**	**0.52 [0.17, 0.87]**	**42**	**0.004**
**Low (< 25 min)**	**9**	**155/139**	**0.45 [0.21, 0.68]**	**0**	**0.0002**
Current density (mA/cm^2^)					
**<0.08**	**9**	**157/147**	**0.48 [0.25, 0.71]**	**11**	**0.0000**
**≥0.08**	**4**	**50/49**	**0.48 [0.07, 0.89]**	**43**	**0.02**
Years of education					
**˃10**	**6**	**68/59**	**0.73 [0.37, 1.10]**	**0**	**0.0000**
**˃6 and ≤10**	**4**	**93/86**	**0.33 [0.04, 0.63]**	**2**	**0.03**
≤ 6	3	40/36	0.38 [−0.09, 0.85]	62	0.11
Cognitive interventions					
**No**	**13**	**152/140**	**0.60 [0.36, 0.84]**	**0**	**0.0000**
Yes	2	73/66	0.21 [−0.12, 0.55]	0	0.21

SMD, standardized mean difference; DLPFC, dorsolateral prefrontal cortex; TL, temporal lobe.

Lines in bold are statistically significant.

### 3.6 Efficacy of tDCS on executive function

**Figure 5:**
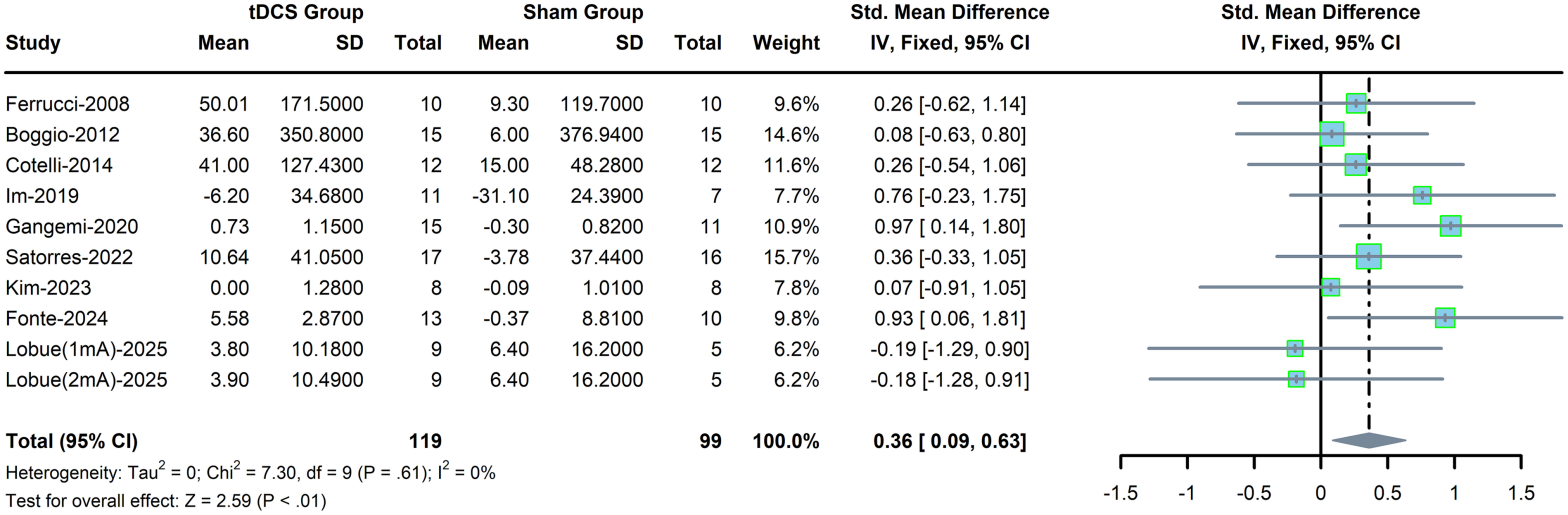
Forest plot for executive function.

Ten trials were included to evaluate the pooled effects of tDCS on executive function in AD patients. A fixed-effects model was selected due to low heterogeneity (*p* = 0.61, *I*^2^ = 0%, Figure 5). The results showed that executive function was significantly enhanced in the tDCS group compared with the sham group (SMD, 0.36; 95% CI, 0.09–0.63; *P*<0.01).

Subgroup analyses revealed that executive function was significantly improved in patients who received stimulation on the L-DLPFC (SMD, 0.46; 95% CI, 0.08–0.84; *p* = 0.02), but not on the TL (SMD, 0.15; 95% CI, −0.40 to 0.71; *p* = 0.59). Additionally, significant improvement was observed in the subgroup with ≤10 tDCS sessions (SMD, 0.44; 95% CI, 0.12–0.77; *p* = 0.007) but not in the subgroup with >10 sessions (SMD, 0.14; 95% CI, −0.37 to 0.66; *p* = 0.58, Table 5). Moreover, patients with 6–10 years of education (SMD, 0.70; 95% CI, 0.22–1.19; *p* = 0.004, Table 5), but not those in the other two education groups, showed improved executive function after tDCS. Similar results were observed in subgroup analyses stratified by current density and concurrent cognitive interventions.

**Table 5:** Results for subgroup analysis of execution.

Subgroup	No. trials	Sample size (tDCS/Sham)	SMD (95% CI)	Heterogeneity (*I*^2^) (%)	*p* value
Stimulation sites					
TL	2	25/25	0.15 [−0.40, 0.71]	0	0.59
**L-DLPFC**	**5**	**61/53**	**0.46 [0.08, 0.84]**	**0**	**0.02**
No. Sessions					
Sessions ˃ 10	4	37/25	0.14 [−0.37, 0.66]	0	0.58
**Sessions ≤10**	**6**	**82/74**	**0.44 [0.12, 0.77]**	**0**	**0.007**
Stimulation time (min)					
High (≥25 min)	3	38/34	0.30 [−0.17, 0.77]	0	0.22
**Low (<25 min)**	**7**	**81/65**	**0.39 [0.06, 0.73]**	**0**	**0.02**
Current density (mA/cm^2^)					
**<0.08**	**5**	**59/51**	**0.41 [0.02, 0.79]**	**0**	**0.04**
**≥0.08**	**3**	**42/38**	**0.48 [0.03, 0.93]**	**0**	**0.04**
Years of education					
˃10	6	68/59	0.13 [−0.23, 0.48]	0	0.48
**˃6 and ≤10**	**3**	**40/33**	**0.70 [0.22, 1.19]**	**0**	**0.004**
≤ 6	1	11/7	0.76 [−0.23, 1.75]	-	0.13
Cognitive interventions					
No	8	94/77	0.30 [−0.00, 0.61]	0	0.05
Yes	1	12/12	0.26 [−0.54, 1.06]	-	0.53

SMD, standardized mean difference; DLPFC, dorsolateral prefrontal cortex; TL, temporal lobe.

Lines in bold are statistically significant.

### 3.7 Efficacy of tDCS on psychological symptoms

**Figure 6:**
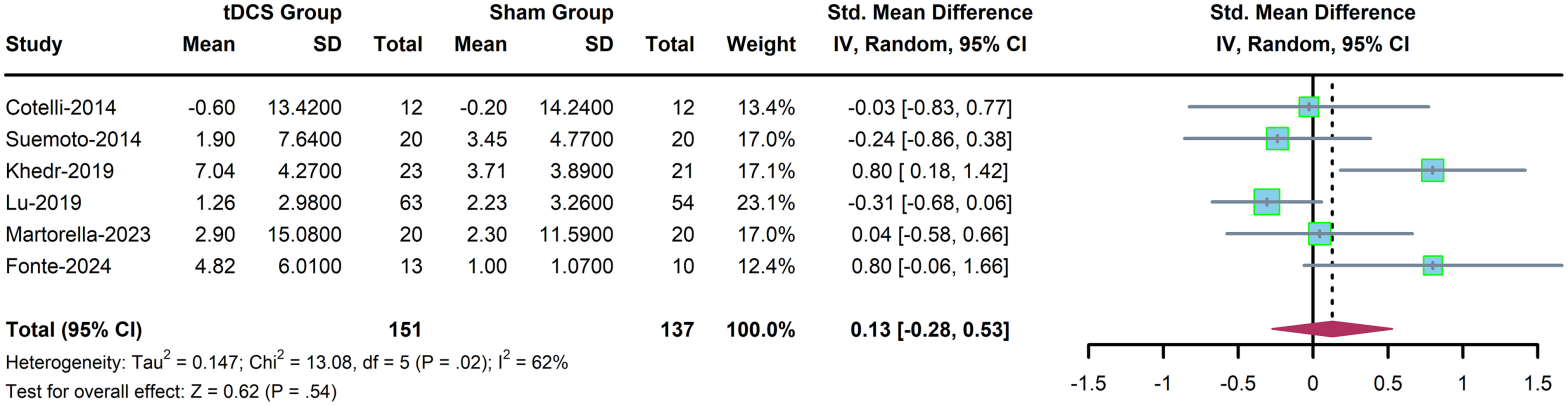
Forest plot for emotional and psychological status.

Six trials were included to summarize the efficacy of tDCS on emotional and psychological symptoms in AD patients. The results did not show a significant difference between the tDCS group and the control group (SMD, 0.13; 95% CI, −0.28 to 0.53; *p* = 0.54, Figure 6). All subgroup analyses in this domain yielded similar results (Table 6), consistent with the findings for language function.

**Table 6:** Results for subgroup analysis of emotional and psychological status.

Subgroup	No. trials	Sample size (tDCS/Sham)	SMD (95% CI)	Heterogeneity (*I*^2^) (%)	*p* value
Stimulation sites					
TL	2	86/75	0.22 [−0.87, 1.30]	89	0.70
L-DLPFC	3	45/42	0.12 [−0.47, 0.72]	47	0.68
No. Sessions					
Sessions ˃ 10	1	63/54	−0.31 [−0.68, 0.06]	—	0.10
Sessions ≤10	5	88/83	0.26 [−0.18, 0.69]	49	0.25
Stimulation time (min)					
High (≥25 min)	2	35/33	0.43 [−0.38, 1.23]	61	0.30
Low (<25 min)	4	116/104	−0.03 [−0.44, 0.37]	48	0.87
Current density (mA/cm^2^)					
<0.08	4	126/115	0.05 [−0.44, 0.53]	69	0.85
≥0.08	2	25/22	0.37 [−0.44, 1.18]	48	0.37
Years of education					
˃10	0	—	—	—	—
˃6 and ≤10	3	88/76	0.07 [−0.57, 0.70]	63	0.84
≤ 6	2	43/41	0.28 [−0.74, 1.30]	81	0.59
Cognitive interventions					
No	3	63/61	0.20 [−0.41, 0.81]	65	0.51
Yes	2	75/66	−0.26 [−0.59, 0.07]	0	0.12

SMD, standardized mean difference; DLPFC, dorsolateral prefrontal cortex; TL, temporal lobe.

### 3.8 Adverse events

Seven of the 23 studies (Hu et al., 2022; Khedr et al., 2014; Khedr et al., 2019; Li et al., 2023; Lu et al., 2019; Smirni et al., 2021; Suemoto et al., 2014) reported adverse events such as headache, blurred vision, itching, and fatigue. Overall, pooling of adverse event data was not feasible due to insufficient descriptions in the original studies. Based on the available data, nearly all adverse events were mild and transient.

### 3.9 Leave-one-out sensitivity analysis

To evaluate the reliability of the meta-analysis results, leave-one-out sensitivity analyses were performed by iteratively excluding one study at a time and recalculating the pooled effect size, significance, and heterogeneity. The significance of the pooled SMD did not change during this process, indicating that the results were not significantly affected by the exclusion of any single study (Supplementary Figures S1–S5). Thus, the results of our meta-analysis were highly robust. Additionally, the leave-one-out approach did not significantly alter the heterogeneity across analyses.

### 3.10 Summary of quality assessment

**Figure 7:**
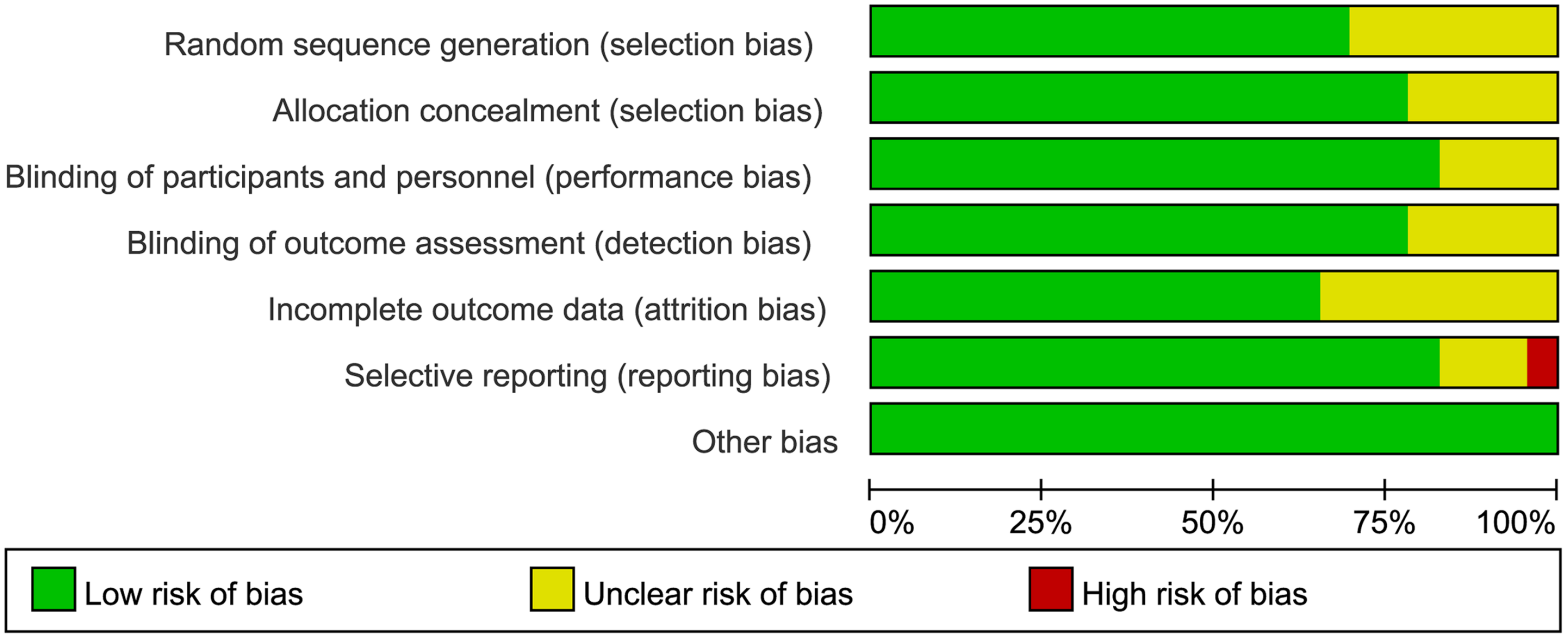
Risk of bias graph.

**Figure 8:**
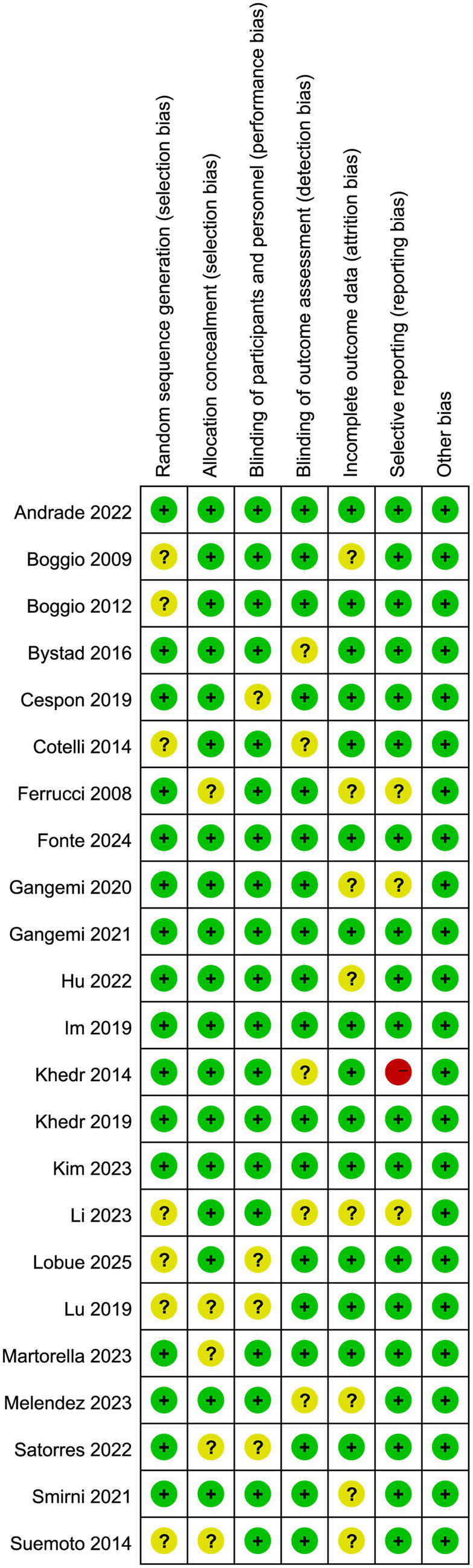
Risk of bias summary.

The RoB2 tool was used to assess the risk of bias in the included studies. All included articles were published in peer-reviewed journals. As shown in Figures 7, 8, all studies employed random allocation. Sixteen studies clearly described the methods for generating the random sequence and were rated as “low risk” (Andrade et al., 2022; Bystad et al., 2016; Cespon et al., 2019; Ferrucci et al., 2008; Fonte et al., 2024; Gangemi et al., 2021; Gangemi and Fabio, 2020; Hu et al., 2022; Im et al., 2019; Khedr et al., 2014; Khedr et al., 2019; Kim and Yang, 2023; Martorella et al., 2023; Meléndez et al., 2023; Satorres et al., 2022; Smirni et al., 2021). In addition, a large proportion of studies implemented allocation concealment, blinding of participants and personnel, and blinding of outcome assessment. These results indicated that the overall quality of the included studies was high. Regarding adverse events, seven studies (Hu et al., 2022; Khedr et al., 2014; Khedr et al., 2019; Li et al., 2023; Lu et al., 2019; Smirni et al., 2021; Suemoto et al., 2014) reported events such as headache, blurred vision, itching, and fatigue. Based on the available data, nearly all adverse events were mild and transient, indicating that tDCS is safe for patients with AD.

### 3.11 Certainty of evidence

The GRADE assessment showed that the certainty of evidence varied across outcomes. The outcomes of global cognition, language, memory, and executive function were rated as moderate quality. The psychological symptom outcome was downgraded to low quality due to serious risk of bias and significant inconsistency. The overall GRADE findings are summarized in Supplementary Table S2.

## 4 Discussion

The present systematic review and meta-analysis evaluated the therapeutic efficacy of transcranial direct current stimulation (tDCS) on cognitive function and psychological status in patients with AD. Compared with our previous study (Wang and Tian, 2024), we included 14 additional original articles in our analyses, doubling the total sample size. Most of these newly added studies were recently published, featuring more rigorous trial designs and more comprehensive patient assessments. Our findings demonstrated that tDCS exerted a positive effect in ameliorating AD-related cognitive dysfunction compared with sham stimulation. When individual cognitive domains were analyzed separately, further subgroup analyses revealed that tDCS significantly improved global cognition, memory, and executive function in AD patients. However, changes in the language domain and psychomotor functions did not reach statistical significance. Overall, the present study further validated the conclusions of our earlier analysis (Wang and Tian, 2024) regarding general cognitive improvement. It also extended the investigation to multiple cognitive and emotional dimensions, including memory, language, executive function, and psychological status, thereby enriching the existing evidence on the effects of tDCS in AD.

Previous studies have indicated that impairment of cortical plasticity in AD patients leads to cognitive dysfunction and declines in activities of daily living (Di Lorenzo et al., 2019; Di Lorenzo et al., 2020). tDCS can modulate neuronal membrane potentials in brain tissue (Kidgell et al., 2013; Kuo et al., 2014; Nitsche et al., 2008), thereby improving patients’ cognitive performance. Although neural plasticity and excitability were not directly measured in our study, the significant improvements in global cognition and specific cognitive domains suggest that tDCS may exert potential regulatory effects on neural plasticity.

Several previous meta-analyses have investigated the impact of tDCS on dementia and cognitive impairment. However, due in part to the limited number of included studies, some of these analyses only summarized the pooled effect of tDCS on overall cognitive function without differentiating distinct cognitive domains (Cai et al., 2019), while others included data from patient populations other than AD (Cruz Gonzalez et al., 2018; Inagawa et al., 2019). For instance, although Cai et al. (2019) reported a favorable effect of tDCS on cognitive performance in AD relative to sham stimulation, they did not explicitly separate different cognitive domains and directly pooled results from multiple distinct outcome measures. Given the increasing application of tDCS in the treatment of AD-related dementia, we deemed it necessary to re-synthesize the current evidence on the efficacy of this technique.

As noted by Wei et al. (2022), cognitive dysfunction in AD encompasses impairments in language, memory, executive function, visuo-spatial function, and other aspects. The present study confirmed significant improvement in global cognitive performance in AD patients following tDCS, which is consistent with the findings of several previous meta-analyses (Majdi et al., 2022; Wang and Tian, 2024). Fifteen of the included studies evaluated global cognitive function using MMSE and ADAS-cog, and nearly all reported similarly positive outcomes (Andrade et al., 2022; Boggio et al., 2012; Cotelli et al., 2014; Fonte et al., 2024; Gangemi et al., 2021; Hu et al., 2022; Im et al., 2019; Khedr et al., 2014; Khedr et al., 2019; Kim and Yang, 2023; Li et al., 2023; Lu et al., 2019; Meléndez et al., 2023; Satorres et al., 2022; Suemoto et al., 2014). Although several individual studies included in our meta-analysis suggested positive effects of tDCS on language function, the pooled results were not statistically significant. This finding is consistent with a previous meta-analysis of repetitive rTMS in AD (Wei et al., 2022). As highlighted by Wei et al. (2022), verbal communication plays a critical role in human social interaction, and language impairment is one of the common early manifestations of AD. The non-significant pooled result for language function may be partially attributed to the heterogeneity of language assessment scales used across studies, including aphasia screening tests, the language subscale of the Wechsler Intelligence Test, the Boston Naming Test, and word fluency tests. This variability hindered the pooling of data and interpretation of results, indicating that further studies are needed to clarify the effects of tDCS on language ability in AD patients.

Consistent with a previous meta-analysis (Majdi et al., 2022), our results showed that tDCS achieved better therapeutic effects than sham stimulation in improving AD-related memory decline. Previous studies have reported a 55% reduction in synapses in early-stage AD patients compared with healthy controls (observed via electron microscopy), and the extent of synaptic loss is correlated with cognitive decline (Coleman and Yao, 2003). tDCS has multifaceted therapeutic potential targeting key AD hallmarks, including synaptic impairment, dopaminergic dysfunction, neuroinflammation, and amyloid plaque deposition (De Paolis et al., 2025). Memory formation is closely associated with brain-derived neurotrophic factor (BDNF), a critical neurotrophic factor involved in dendrite growth and neuronal development (Banerjee and Shenoy, 2023), and decreased BDNF levels are correlated with memory decline in AD patients (Lim et al., 2021). A recently published study (Chen et al., 2025) indicated that tDCS may exert neuroprotective effects by upregulating key downstream molecules of the BDNF signaling pathway, thereby alleviating memory decline and promoting functional recovery. Regarding executive function, our results showed a significant improvement in the tDCS group compared with the control group, which was consistent with the pooled effect observed for memory.

With respect to psychological symptoms, our results revealed no significant difference between the tDCS and control groups. A previous meta-analysis (Wang et al., 2020b) investigating the role of non-invasive brain stimulation (NIBS) in behavioral and psychological symptoms of AD also found that, in contrast to rTMS, tDCS did not improve psychological deficits post-treatment. Overall, evidence regarding the efficacy of tDCS on psychological symptoms in AD remains limited and controversial. Therefore, more well-designed randomized controlled trials are needed before definitive conclusions can be drawn.

In theory, the response to tDCS in AD patients is influenced by multiple factors, including: the cognitive or psychological domain being evaluated (e.g., language, memory, executive function); individual characteristics (e.g., skull thickness, subcutaneous fat, brain anatomical heterogeneity, and baseline factors such as age and education level); and tDCS parameters (e.g., current density, target region, number of sessions, and stimulation duration) (Majdi et al., 2022). To address this, we performed subgroup analyses to compare different tDCS settings and identify potential candidates who may benefit more from this intervention.

Controversies exist regarding the optimal stimulation target for tDCS in AD. Among the included original studies, DLPFC was the most commonly selected target. This preference may be attributed to the critical role of the DLPFC in cognitive function. Previous studies have identified the DLPFC as a compensatory brain region associated with cognition, and its compensatory capacity declines with AD progression (Gigi et al., 2010). Additionally, disrupted functional connectivity of the DLPFC in individuals with cognitive impairment negatively affects multiple cognitive domains (Liang et al., 2011). Meléndez et al. (2023) reported significant improvements in MMSE scores, immediate memory, and delayed recall following 5 sessions of tDCS targeting the DLPFC. Another study by Fonte et al. (2024) found that anodal tDCS of the DLPFC combined with motor or cognitive training improved global cognition, attention, and slowed cognitive decline in AD patients. Furthermore, Satorres et al. (2022) demonstrated significant improvements in global cognition, immediate and delayed memory, and learning ability in the active tDCS group targeting the DLPFC. However, a previous meta-analysis (Wang and Tian, 2024) reported significant cognitive improvement in AD patients receiving tDCS targeting the TL, rather than L-DLPFC. Majdi et al. (2022) found no significant differences between TL and L-DLPFC tDCS in terms of memory, attention, or general cognition. In contrast, a meta-analysis of rTMS showed that stimulation of the DLPFC, but not the TL, significantly improved cognition (Zhang et al., 2022). Our study indicated that both L-DLPFC and TL tDCS improved global cognition and memory; however, significant improvement in executive function was only observed in patients receiving L-DLPFC stimulation, not TL stimulation.

We attempted to interpret these controversies from multiple perspectives. First, there is substantial methodological heterogeneity across the original trials. Second, the use of diverse outcome measures and assessment scales makes it difficult to synthesize results based on a standardized criterion. Third, the differing results may also be partially attributed to the distinct mechanisms of action between tDCS and rTMS. rTMS induces neuronal depolarization via electromagnetic induction of a weak electric field in the cortex (Teselink et al., 2021), whereas tDCS modulates cortical excitability through a constant weak current delivered by scalp electrodes. Overall, whether the DLPFC is superior to other regions as a stimulation target, and the optimal target for distinct cognitive domains, requires further investigation.

Controversies also exist regarding optimal tDCS parameters. For example, regarding the number of tDCS sessions, Cai et al. (2019) suggested that a single session of tDCS is more effective than multiple sessions, while another study found significant improvements in memory and attention only after repeated tDCS sessions (Majdi et al., 2022). However, in our meta-analysis, only three studies applied a single session of tDCS (Cespon et al., 2019; Ferrucci et al., 2008; Smirni et al., 2021). Therefore, consistent with our previous study (Wang and Tian, 2024), we used a cutoff value of 10 sessions based on the distribution of session numbers across included studies. Similar to our previous findings (Wang and Tian, 2024), we found no significant difference in global cognition between patients receiving >10 sessions and those receiving ≤10 sessions. Additionally, we observed significant improvements in memory and executive function in patients receiving ≤10 sessions of tDCS. Similarly, our results indicated that stimulation duration <25 min was superior to ≥25 min in improving executive function. The biological mechanism underlying this phenomenon remains unclear. In theory, additional neuromodulation sessions would be expected to yield greater benefits. We speculate that the number of tDCS sessions is not linearly associated with therapeutic efficacy; instead, there may be an optimal threshold. Excessive stimulation may increase the neuronal excitation threshold, thereby weakening the therapeutic effect, leading to reduced performance in patients receiving more sessions than this threshold. However, the optimal number of tDCS sessions and the precise mechanism underlying these findings require further research. Regarding current density, our previous study (Wang and Tian, 2024) showed that a current density of ≥0.08 mA/cm^2^ was superior to <0.08 mA/cm^2^ in improving global cognition. However, our subgroup analyses in the present study found no significant differences in any cognitive domain based on current density. Although a higher current density tends to induce greater cortical excitability (Bastani and Jaberzadeh, 2013), excitability level does not directly translate to greater cognitive improvement, given the multiple influential factors mentioned earlier.

Our subgroup analyses further indicated that tDCS alone (without CT) alleviated memory decline in AD patients. This result is consistent with our previous finding regarding global cognition (Wang and Tian, 2024). Several studies involving rTMS (Lin et al., 2019; Zhang et al., 2022) also reported that rTMS alone was superior to rTMS combined with CT. These results may be partially explained by the hypothesis that NIBS and CT do not exert synergistic effects. However, the actual effect of concurrent cognitive intervention and the underlying neural mechanisms require further investigation.

Regarding patient individual factors, significant improvement in executive function was observed in patients with a relatively high education level (>6 years), but not in the subgroup with 6–10 years of education. The neural mechanism underlying this observation remains largely unknown. It has been suggested that education level is associated with cognitive reserve, and individuals with higher education are more capable of recruiting cognitive-related brain networks (Cai et al., 2019; Stern, 2002). However, it should be noted that higher cognitive reserve may not always directly translate to greater improvements in all cognitive domains (e.g., memory and global cognition). Education level is recommended as a potential modulator of treatment response, but not a determinant of tDCS efficacy. Furthermore, the current observation is preliminary, and thus the effects of education level should be interpreted with caution, requiring further validation in future studies.

In addition to the aforementioned key findings, changes in electroencephalographic (EEG) signals represent another important outcome measure that warrants discussion. A total of six included articles reported EEG results (Andrade et al., 2022; Cespon et al., 2019; Cotelli et al., 2014; Gangemi et al., 2021; Gangemi and Fabio, 2020; Li et al., 2023). Overall, the EEG findings were controversial. Cotelli et al. (2014) reported that tDCS reduced P300 latency but had no effect on motor cortex excitability. Another study (Cespon et al., 2019) found a correlation between improved working memory after anodal tDCS and increased P300 amplitude in healthy elderly individuals, but not in AD patients. In contrast, Gangemi and Fabio (2020) demonstrated that tDCS improved cognitive function, increased beta band activity, and reduced P300 latency in AD patients. None of the included studies reported magnetic resonance imaging (MRI)-related findings following tDCS. Therefore, the effects of tDCS on EEG and MRI parameters require further investigation.

The present study included recently published articles based on explicit inclusion criteria, analyzed diverse functional domains separately, and performed subgroup analyses to identify potential factors influencing tDCS efficacy in AD. Leave-one-out sensitivity analyses confirmed the high reliability of our results. Adverse events and dropout rates were also summarized for each original study. To the best of our knowledge, this is the most comprehensive study to date evaluating the pooled effects of tDCS on distinct functional domains in AD. Although tDCS is currently considered an investigational intervention, our study enriches the existing literature and holds promise for optimizing this technique to ultimately facilitate the treatment of AD.

This study has several limitations. First, substantial methodological heterogeneity existed across the included original studies, such as differences in current density and electrode placement, leading to high heterogeneity in the meta-analysis. Leave-one-out sensitivity analyses did not significantly reduce this heterogeneity, suggesting that the high heterogeneity is primarily attributable to methodological differences across studies rather than outliers. Second, although 23 articles were included, the sample size in some subgroup analyses remained relatively small, which may have limited the statistical power of these analyses. Third, the exact mechanisms underlying some of our findings (e.g., the effect of education level) are difficult to interpret, and most of our interpretations are based on hypotheses rather than direct experimental evidence. Fourth, nearly all included studies focused on patients with mild to moderate AD; thus, the efficacy of tDCS in severe AD remains unclear and requires further research.

## 5 Conclusion

In conclusion, the present systematic review and meta-analysis demonstrated that tDCS improves global cognition, memory, and executive function in AD patients, but does not significantly affect language function or psychomotor symptoms. However, due to the relatively high heterogeneity of the included data, our results should be interpreted with caution. Further well-designed, large-scale randomized controlled trials are warranted to validate these findings before tDCS could be established as a standard therapeutic intervention for AD.

## Data Availability

The original contributions presented in the study are included in the article/Supplementary material, further inquiries can be directed to the corresponding author.

## Supplementary material

SUPPLEMENTARY FIGURE S1 Sensitivity analysis for global cognition.

SUPPLEMENTARY FIGURE S2 Sensitivity analysis for language ability.

SUPPLEMENTARY FIGURE S3 Sensitivity analysis for memory.

SUPPLEMENTARY FIGURE S4 Sensitivity analysis for executive function.

SUPPLEMENTARY FIGURE S5 Sensitivity analysis for emotional and psychological status.
