# Supplementary material for: The therapeutic efficacy of transcranial direct current stimulation in managing Alzheimer’s disease: a systematic review and meta-analysis
Source: Front Aging Neurosci. 2026 Apr 10;18:1726469. doi: 10.3389/fnagi.2026.1726469 (PMC13106126; doi:10.3389/fnagi.2026.1726469)
Supplement: Supplementary file 7 [file Data_Sheet_2.pdf]

Table S2. Summary of GRADE rating

Outcome	No. of Patients (studies)	Effect size (SMD, 95% CI)	Risk of bias	Inconsistency	Indirectness	Imprecision	Other considerations[a]	Overall certainty of evidence
General Cognition	640 (16 RCTs)	0.66 (0.38-0.93)	Serious[b]	Not serious	Not serious	Not serious	Not serious	⊕⊕⊕⊖ **moderate**
Language	336 (10 RCTs)	0.22 (0-0.44)	Serious[b]	Not serious	Not serious	Not serious	Not serious	⊕⊕⊕⊖ **moderate**
Memory	431 (15 RCTs)	0.47 (0.27-0.66)	Not serious	Serious[c]	Not serious	Not serious	Not serious	⊕⊕⊕⊖ **moderate**
Executive function	218 (10 RCTs)	0.36 (0.09-0.63)	Serious[b]	Not serious	Not serious	Not serious	Not serious	⊕⊕⊕⊖ **moderate**
Emotion	288 (6 RCTs)	0.13 (-0.28-0.53)	Serious[b]	Serious[c]	Not serious	Not serious	Serious	⊕⊕⊖⊖ **low**[1]

RCT = randomized controlled trials.

[a] Other considerations are publication bias, large effect, dose response, and plausible confounding factors.

[b] As the outcome had significant methodological quality concerns.

[c] As the outcome had significant heterogeneity

Moderate quality: Further research is likely to have an important impact on our confidence in the estimate of effect and may change the estimate.

Low quality: Further research is very likely to have an important impact on our confidence in the estimate of effect and is likely to change the estimate.
